# Mink infection with influenza A viruses: an ignored intermediate host?

**DOI:** 10.1186/s44280-023-00004-0

**Published:** 2023-03-30

**Authors:** Chris Ka Pun Mok, Kun Qin

**Affiliations:** 1 The Jockey Club School of Public Health and Primary Care, The Chinese University of Hong Kong, SAR Hong Kong, China; 2 Li Ka Shing Institute of Health Sciences, Faculty of Medicine, The Chinese University of Hong Kong, SAR Hong Kong, China; 3 National Institute for Viral Disease Control and Prevention, Chinese Center for Disease Control and Prevention (China CDC), 100 Yingxin Street, Western District, 100052 Beijing, China

**Keywords:** Mink, Influenza, Pandemic

## Abstract

Continuously emergence of human infection with avian influenza A virus poses persistent threat to public health, as illustrated in zoonotic H5N1/6 and H7N9 infections. The recent surge of infection to farmed mink by multiple subtypes of avian influenza A viruses in China highlights the role of mink in the ecology of influenza in this region. Serologic studies suggested that farmed mink in China are frequently infected with prevailing human (H3N2 and H1N1/pdm) and avian (H7N9, H5N6, and H9N2) influenza A viruses. Moreover, genetic analysis from the sequences of influenza viruses from mink showed that several strains acquired mammalian adaptive mutations compared to their avian counterparts. The transmission of SARS-CoV-2 from mink to human alerts us that mink may serve as an intermediate host or reservoir of some emerging pathogens. Considering the high susceptibility to different influenza A viruses, it is possible that mink in endemic regions may play a role as an “mixing vessel” for generating novel pandemic strain. Thus, enhanced surveillance of influenza viruses in mink should be urgently implemented for early warning of potential pandemic.

## Current situation of influenza A virus infection in mink

**Fig. 1:**
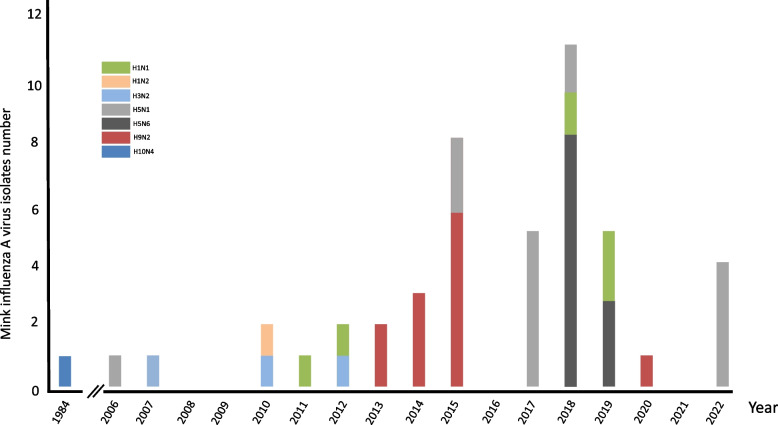
Mink influenza A virus detected worldwide annually. A total of 47 influenza viral sequences were obtained from GISAID, consisting of seven subtypes, H1N1, H1N2, H3N2, H5N1, H5N6, H9N2 as well as H10N4 from year 1984 to 2022. Most of the mink influenza A viruses were detected from China since 2013, with the majority comprising of H9N2 and H5N6. During the pandemic of SARS-CoV-2 from 2020-present, fewer mink influenza virus was submitted and reported

Influenza A viruses are susceptible to a wide range of host including avian species, human and marine mammals. The species jumping, especially from avian to human, resulted to the occurrence of at least four pandemic in human history [1]. Frequent intersperse transmissions and further adaptation facilitated the emergence of avian influenza viruses in human. Although aquatic birds was widely accepted as the natural reservoir for influenza A virus, outbreak of highly pathogenic avian influenza virus (HPAIV) H5N1 in migratory waterfowl in Qinghai suggested that continuing evolution of influenza A virus may drive them expand their host range [2]. Infection under laboratory setting demonstrated that several influenza A subtypes could infect mink and transmit the virus to the close contacts via aerosol [3–6]. Since infection with avian influenza A virus (H10N4) was first found in Sweden in 1984 [7–10], infections in mink by different influenza subtypes such as avian H5N1, H5N6, H9N2, as well as human/swine H1N1 and H3N2 viruses were reported worldwide [11–23]. Indeed, available sequence data from GISAID revealed that there is an increasing detection rate of avian influenza A virus in mink during the past decade (Fig. 1). For instances, mink infection with avian H9N2 influenza A virus was reported in 2013 in China and infections of avian H5N1 and H5N6 were also documented frequently [16, 17] (Table 1). European avian-like (EA-like) swine H1N1 virus was reported to infect mink in 2018 [12]. Moreover, swine-origin H1N1/2 and H3N2 variants were found from diseased mink in Canada and USA during 2007 to 2012 [19–23]. When the H1N1/pdm successfully replaced the circulating seasonal strain in human in 2009, it was also found in mink in Europe, North America and China [19, 24] . Although well defined H1, H3 and H7 lineages of influenza variants have been dominating in pig, horse and canine respectively for long time [25], no specific subtype or lineage has been well established in farmed mink population. However, the permissiveness of mink to be infected by diverse subtypes of influenza A virus from either avian or human/swine origin may facilitate the reassorment process, generating novel strain with pandemic potential.

**Table 1:** Summary of influenza A viruses detected from mink. All mink influenza viral sequences were obtained from GISAID. Most viruses contained entire viral genomic sequence with 8 segments, while some viruses had partial sequences available. Most viral lineage was defined using the full HA sequence of each mink virus to determine the phylogenic relationship with reference sequences according to WHO guideline. Due to unavailability of some viral sequences of PB2 gene, adaptive mutations at 627 and 701 amino acid position of these virus can not be examined and analyzed

Strain	Subtype	Region^a^	Seq availability^b^	Most related viral lineage^c^	PB2 mutation	
					627	701
A/mink/Sweden/E12665/1984	H10N4	EU	NA	Wild bird H10 virus	-^d^	–
A/mink/Sweden/V907/2006	H5N1	EU	ALL	QH-like HPAIV H5/cl.2.2	**K**	D
A/mink/Nova Scotia/1055488/2007	H3N2	NA	ALL	Triple reassortant swine H3	E	D
A/mink/Nova Scotia/1265707/2010	H3N2	NA	HA/M	Hybrid of swine H3 and H1N1/pdm	–	–
A/mink/Iowa/30943/2010	H1N2	NA	M	Natural occuring H1N2	–	–
A/Mink/Netherlands/11009347/2011	H1N1	EU	NA	H1N1/pdm	–	–
A/mink/Wisconsin/31512–7/2012	H3N2	NA	ALL	Local swine H3N2 virus	E	D
A/mink/South Dakota/A01279427/2012	H1N1	NA	ALL	Local swine H1N1 virus	E	D
A/mink/Shandong/F6/2013	H9N2	China	ALL	Local avian H9N2 virus/Y280	**K**	D
A/mink/Shandong/F10/2013	H9N2	China	ALL	Local avian H9N2 virus/Y280	**K**	D
A/mink/China/01/2014	H9N2	China	ALL	Local avian H9N2 virus/Y280	E	**N**
A/mink/China/02/2014	H9N2	China	ALL	Local avian H9N2 virus/Y280	E	D
A/mink/Shandong/WM01/2014	H9N2	China	ALL	Local avian H9N2 virus/Y280	E	**N**
A/mink/China/XB/2015	H5N1	China	ALL	Local avian H5N1 virus/cl.2.3.2.1e	E	D
A/mink/China/G/2015	H5N1	China	ALL	Local avian H5N1 virus/cl.2.3.2.1b	E	D
A/mink/Shandong/Z1/2015	H9N2	China	ALL	Local avian H9N2 virus/Y280	E	**N**
A/mink/Shandong/Z2/2015	H9N2	China	ALL	Local avian H9N2 virus/Y280	E	**N**
A/mink/Shandong/Z3/2015	H9N2	China	ALL	Local avian H9N2 virus/Y280	E	**N**
A/mink/Shandong/Z4/2015	H9N2	China	ALL	Local avian H9N2 virus/Y280	E	**N**
A/mink/Shandong/Z5/2015	H9N2	China	ALL	Local avian H9N2 virus/Y280	E	**N**
A/mink/Shandong/Z6/2015	H9N2	China	ALL	Local avian H9N2 virus/Y280	E	**N**
A/mink/China/CY/2017	H5N1	China	ALL	Local avian H5N1 virus/cl.2.3.2.1e	E	D
A/mink/China/CY1/2017	H5N1	China	HA/NP/NA	Local avian H5N1 virus/cl.2.3.2.1e	–	–
A/mink/China/CY2/2017	H5N1	China	HA/NP/NA	Local avian H5N1 virus/cl.2.3.2.1e	–	–
A/mink/China/CY3/2017	H5N1	China	HA/NP/NA	Local avian H5N1 virus/cl.2.3.2.1e	–	–
A/mink/China/CY4/2017	H5N1	China	HA/NP/NA	Local avian H5N1 virus/cl.2.3.2.1e	–	–
A/mink/Shandong/1121/2018	H1N1	China	ALL	Local swine H1N1 virus/EA-H1N1	E	D
A/mink/Eastern China/006/2018	H5N6	China	ALL	Local avian H5N6 virus/cl.2.3.4.4	E	D
A/mink/Eastern China/032/2018	H5N6	China	ALL	Local avian H5N6 virus/cl.2.3.4.4	E	D
A/mink/Northern China/110/2018	H5N6	China	ALL	Local avian H5N6 virus/cl.2.3.4.4	E	D
A/mink/Eastern China/149/2018	H5N6	China	ALL	Local avian H5N6 virus/cl.2.3.4.4	E	**N**
A/mink/Eastern China/528/2018	H5N6	China	ALL	Local avian H5N6 virus/cl.2.3.4.4	E	**N**
A/mink/Eastern China/0824/2018	H5N6	China	ALL	Local avian H5N6 virus/cl.2.3.4.4	**K**	D
A/mink/Eastern China/0712/2018	H5N1	China	ALL	Local avian H5N1 virus/cl.2.3.4.4	E	D
A/mink/Eastern China/571/2018	H5N6	China	ALL	Local avian H5N6 virus/cl.2.3.4.4	E	D
A/mink/Northern China/F0130m/2018	H5N6	China	N/M/NS	–	–	–
A/mink/China/456/2018	H5N6	China	ALL	Local avian H5N6 virus/cl.2.3.4.4	E	D
A/mink/Utah/19–015857-001/2019	H1N1	NA	ALL	H1N1/pdm	E	D
A/mink/China/0509/2019	H1N1	China	ALL	H1N1/pdm	E	D
A/mink/China/181/2019	H5N6	China	ALL	Local avian H5N6 virus/cl.2.3.4.4	**K**	D
A/mink/China/183/2019	H5N6	China	ALL	Local avian H5N6 virus/cl.2.3.4.4	**K**	D
A/mink/China/191/2019	H5N6	China	ALL	Local avian H5N6 virus/cl.2.3.4.4	**K**	D
A/mink/China/chick embryo/2020	H9N2	China	ALL	Local avian H9N2 virus/ Y280	V	D
A/mink/Spain/3691-8_22VIR10586–10/2022	H5N1	EU	ALL	Local avian H5N1 virus/cl.2.3.4.4	E	D
A/mink/Spain/3691-10_22VIR10586–11/2022	H5N1	EU	ALL	Local avian H5N1 virus/cl.2.3.4.4	E	D
A/mink/Spain/3691–2_22VIR10586–8/2022	H5N1	EU	ALL	Local avian H5N1 virus/cl.2.3.4.4	E	D
A/mink/Spain/3691–3_22VIR10586–9/2022	H5N1	EU	ALL	Local avian H5N1 virus/cl.2.3.4.4	E	D

^a^EU represents European Union; NA represents North America

^b^Influenza A viruses genome consists of eight gene segments: PB2, PB1, PA, HA, NP, NA, M, NS. All available viral sequence from GISAID were downloaded and analyzed

^c^Mink influenza viruses were classified within HA serotype using the full length of HA sequence (1-1649 nt of H1, H3, H5, H7, H9, H10), if applicable. All clade and lineage were defined according to WHO guideline. Viral genome were aligned with reference sequences and phylogenically analyzed using Mega software. “-” indicates the unavailability of the HA sequence for analysis

^d^Residue with mammalian adaptation mutations were in bold. “-” indicates the unavailability of the PB2 sequence for analysis

Mink (*Mustela vison*), belonging to mustelidae, is a small fur-bearing animal with a high economic value [17]. Although experimental studies have demonstrated a high susceptibility of mink infection by diverse influenza A viruses, outbreaks in mink population associated with the development of severe clinical signs in infected animals is a rare event [23]. Ferret, which is one of the closest relatives of mink, is used as the animal model to study influenza virus, due to its high susceptibility to the virus and similar pathobiology to humans after infection [26]. Studies revealed that the animal feed which was composed of raw poultry by-products and pork meat were mainly responsible for infection of avian or swine influenza A virus in mink. On the other hand, infection with human influenza virus was likely transmitted by the farm workers [3, 24]. In general, mink showed mild to moderate respiratory symptoms after being infected by influenza virus [3]. Interestingly, co-infections of influenza virus with other respiratory pathogens were reported in mink with self-limited respiratory symptoms [12]. The transmission chains of avian influenza A virus from poultry to mink could be immediately blocked by stopping feeding poultry products contaminated with flu virus. Therefore, less attention was paid on the influenza infection in mink compared to commonly found canine distemper virus (CDV) and Aleutian mink disease virus (AMDV).

## Threats posed by mink with emerging viral infection

Multiple outbreaks in mink farms which were caused by other viruses were reported in China since 2010. In 2011, a new orthoreovirus was identified in a farm from Hebei, with an estimated mortality of < 5% [27]. An invasive outbreak of swine pseudorabies was reported in 2014 in a mink farm from Shandong, with a mortality rate of 87% [28]. Newcastle disease in mink from Heilongjiang in 2014 was found and was responsible for the hemorrhagic encephalitis and pneumonia, with a death rate of 95% in the 9% of affected mink [29]. In 2015, outbreak with HPAIV H5N1 influenza virus were reported in two mink farms in Northeast China, with the death rates of 56 and 64%, respectively [16]. Since the emergence of SARS-CoV-2 in Wuhan in late 2019, farmed mink infections with this strain have been repeatedly reported worldwide, especially in Europe and North American countries [30, 31]. Furthermore, transmissions of SARS-CoV-2 from mink to human were reported in Netherlands and Denmark [32, 33]. It should be emphasized that many viruses found in mink are known to have potential causing pandemic in human.

It was suggested that the reassortment and adaptation are two key mechanisms for the emergence of zoonotic influenza virus in human [1]. There is a concern that co-infection of animal and human hosts with different viruses in the same host may generate novel strain with pandemic potential. Pigs have been regarded as an ideal “mixing vessel” for generating pandemic strain and as they express receptors that favor the binding of both avian and human influenza viruses [34]. Similar condition is also found in mink. The sialic acid receptors, SA-α-2,3-Gal and SA-α-2,6-Gal, are both found in the respiratory tracts of mink [3]. Thus, it is postulated that frequent interspecies transmissions of different subtype viruses in mink under natural condition will increase the chance of generating novel strain during co-infection of two different subtypes. Given that multiple subtypes of avian influenza viruses are prevalent in Southeast Asia, mink may be recognized as another “mixing vessel” to generate novel strain or serve as an “amplifier” in the transmission process.

## Mink farming and common infectious diseases

**Fig. 2:**
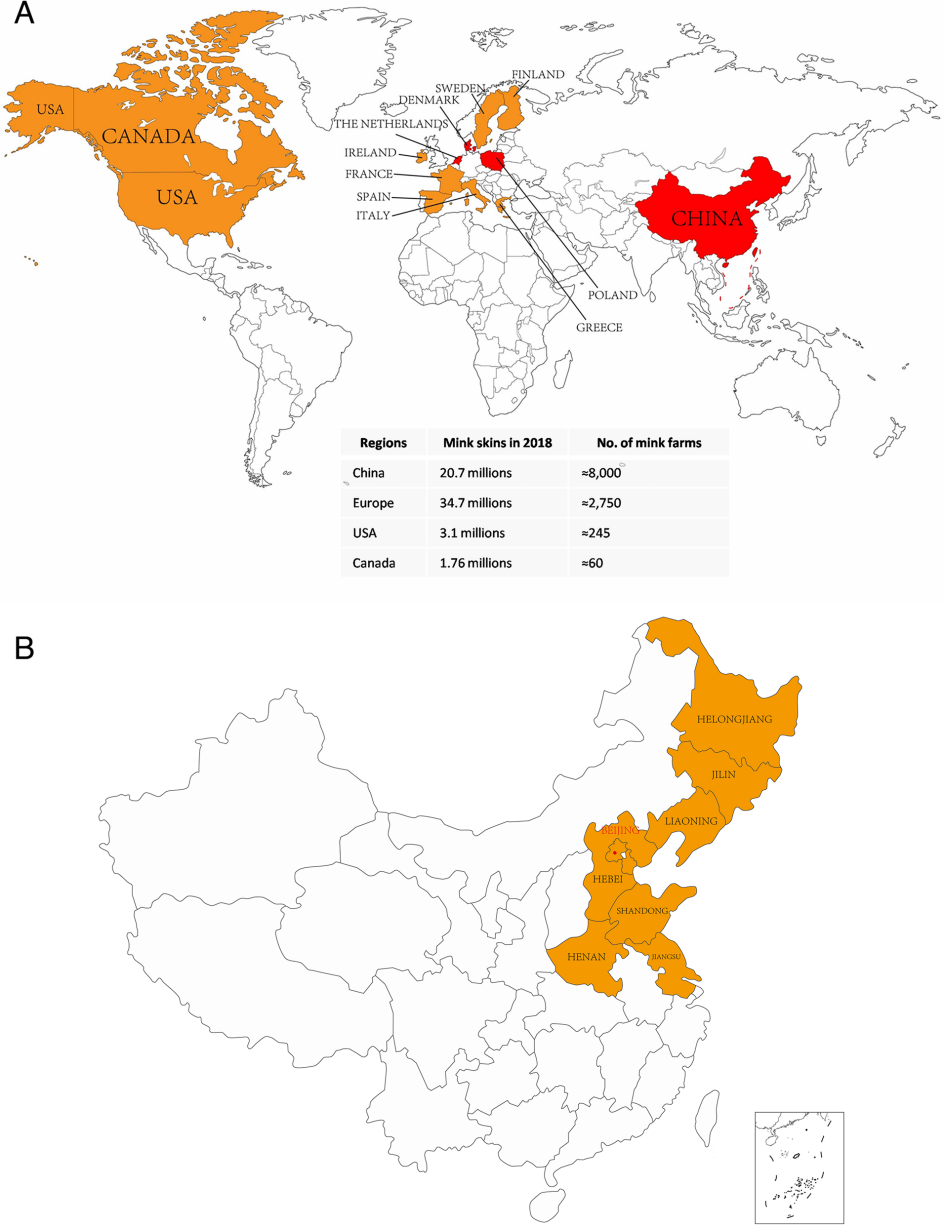
(**A**) Distribution of major mink farms worldwide. Data derived from Fenollar F et al. [30], China, Denmark, Netherlands as well as Poland (red in the map) are the four major fur-production countries. (**B**) Distribution of mink farms in China

The American mink (*Neovison vison*) is currently the most important species in fur-farming industry [35]. The main fur producing countries are Denmark, Netherlands, Poland, and China (Fig. 2A). China is the leading market for fur and the first mink farms were established in the 1950s. Currently, the annual production of Chinese mink is over 20 million (20.7million minks in 2018), representing 23.8% of the world’s market share [30, 36]. Thus, China is a major player in the mink farming industry which is still expanding rapidly. The major mink farming areas in China are located in the northern and northeast areas where the climate is ideal and the feed supply is abundant [39]. About 80% of mink are farmed in Shandong Province [37] (Fig. 2B).

In those large farms, minks are caged in high density and it is common that they are housed in a single shelter or building. Infectious disease usually occurred due to intensive farming practices, poor sanitation and insufficient biosecurity practise. For instances, AMDV is the most important viral infection for mink because of the devastating threat to adults’ reproduction system and the subsequent quality of the fur [36]. Respiratory and gastroenteritic symptoms are mainly caused by influenza A virus, CDV, mink enteritis virus (MEV) as well as recently recognized SARS-CoV-2 [30]. Besides, several bacterial has been recognized to cause hemorrhagic pneumonia in mink, such as *Pseudomonas aeruginosa, Klebsiella pneumoniae* as well as *Escherichia coli* [38].

Several subtypes of influenza A virus are prevalent since 1990s in domestic poultry in China [39]. Establishment of multiple genotypes of H9N2 and H5 HPAI variants in this region contributes to the emergence of novel H7N9 and H10N8 viruses which claimed 621 human lives [40, 41]. Infections of poultry with these variants have been frequently reported although compulsory immunization strategy is implemented in China [39]. As poultry and by-products (head, bones, offal) with dry form are essential components in mink feed, transmissions of influenza viruses from poultry to mink via contaminated poultry are inevitable. Moreover, transportation of minks from breeding farms to private or family-run farms also contribute to the disease dissemination.

## Molecular characterization of influenza viruses that are identified in mink

Available sequences from GISAID revealed that all 47 influenza A viruses found in mink are phylogenetically related to prevailing strains from poultry, wild birds, swine, as well as human (Table 1). Among them, H9N2 and HPAIV H5N6/N1 were the two predominant subtypes in mink in China since 2013. In addition, European avian like (EA-like) swine H1N1 and pdm/H1N1 viruses which are prevalent in swine and human in China were also found in mink. Genetic analysis showed that all the H9N2 viruses found in mink are belonged to the G9/Y280 lineage which is prevalent in poultry in this region. Variants from this lineage was also responsible for recent surge of human infection [39]. Except for one HPAIV H5N1 (belonged to the Qinghai lineage (Clade 2.2.1)) was detected in mink in Sweden in 2006 and was believed to be transmitted by migrating bird, all other H5N1 and H5N6 subtypes detected from China and Spain were likely introduced from infected poultry. Seven H5N1 viruses identified from 2015 to 2017 were of clade 2.3.2.1 and other H5 subtypes of influenza virus detected since 2018 (H5N1 and H5N6) from China and Europe were belonging to clade 2.3.4.4 (Table 1).

Molecular analysis showed that some mammalian adaptive signatures such as E627K and D701N mutations could be found in the PB2 sequence that these mutations can support the virus in replicating highly in mammals [42, 43]. All type A influenza viruses from avian hosts contained E at 627 amino acid position in PB2, except for those of Qinghai-lineage H5N1 which caused the first outbreak in migratory birds and subsequently transmit to European and Africa regions [2]. While the early H5N1 isolate (A/mink/Sweden/V907/2006) in mink carried 627 K directly from wild birds or affected domestic poultry, four Chinese H5 isolates from mink (A/mink/Eastern China/0824/2018, A/mink/China/181/2019, A/mink/China/183/2019, A/mink/China/191/2019) possessed K at the 627 amino acid in PB2. In addition, two mink H9N2 (A/mink/Shandong/F6/2013 and A/mink/Shandong/F10/2013) viruses detected in 2013 from China also contained K at this position. It was noted that D to N mutation at 701 position of PB2 can be observed in the most of Chinese H9N2 (8 out of 12) and two H5N6 isolates. However, co-mutations (E627K and D701N mutations in PB2 detected simultaneously in one virus) was not present in both mink H5 and H9 isolates. Long-term prevalence of H5 and H9 influenza A viruses in Chinese poultry seems to prevent the introduction and establishment of Qinghai-lineage variant in this region. Thus, it is important to monitor the occurrence of these mammalian host specific mutations in mink.

## Serology of influenza virus infection in mink

There is currently no influenza vaccination program for mink in China, serological investigations could provide insight into the infection status of influenza A virus in mink population. Since the first isolation of mink H9N2 influenza in 2013 in Shandong, multiple studies have been performed to determine the sero-prevalence of avian influenza A virus infection in mink [3, 15, 17, 18, 44–46] (Table 2). In general, the seropositive rate against H9N2 ranked the highest among all subtype infections in mink, ranging from 20 to 47.5% during 2013 to 2019. Compared to H9N2, the positive rates of H5N1 and H5N6 were much lower, at 6.7% using clade 7.2 virus and 2.8% with clade 2.3.4 virus as the antigen, respectively. Since the outbreak of H7N9 in humans in China in 2013, 6.9% and 3.7% of serum samples collected from eastern and northern China, respectively, showed positive for this virus. Sera collected from mink slaughterhouses during 2016 to 2019 from Shandong and Hebei revealed that 47.3 and 11.4% were seropositive against human seasonal pdmH1N1 and H3N2 viruses respectively. These sero-epidemiological data strongly suggested that farmed mink in China were highly exposed to both avian and human influenza viruses, increasing the likelihood of co-infection with variants from different origins.

**Table 2:** Serology survey of influenza A virus infection in mink. Multiple serology studies were performed to determine the status of influenza infection in mink population mostly in China by different individual research groups. Due to the large number mink farms located in Shandong province, the majority of the studies were focusing on the mink from this region

Antigen^a^	Sample information^b^	Regions/Provinces	Positive rate (%)	Reference
H1N1	Slaughter plant, 2016–2019	Shangdong & Hebei	47.3	Sun H et al. 2021, EMI [3]
H3N2	Slaughter plant, 2016–2019	Shangdong & Hebei	11.4	Sun H et al. 2021, EMI [3]
H5N1	Healthy mink farm, 2013	Shandong	6.7	Zhang C et al. 2015, Viro J [17]
H5N6	Slaughter plant, 2016–2019	Shangdong & Hebei	2.8	Sun H et al. 2021, EMI [3]
H7N9	Healthy mink farm, 2017	Eastern China	6.9	Yu Z et al. 2020, Braz J Microbiol [38]
	Slaughter plant, 2016–2019	Shangdong & Hebei	3.7	Sun H et al. 2021, EMI [3]
H9N2	Resp.diseased farm, 2013	Shandong	20	Li P et al. 2015, Vet Microbiol [18]
	Healthy mink farm, 2017	Eastern China	57	Yu Z et al. 2020, Braz J Microbiol [38]
	Healthy mink farm, 2015	Shandong	31	Zhao Y et al. 2017, Sci Rep [29]
	Healthy mink farm, 2013	Shandong	47.5	Zhang C et al. 2015, Virol J [17]
	Slaughter plant, 2016–2019	Shangdong & Hebei	39.7	Sun H et al. 2021, EMI [3]

^a^Influenza viruses were used as antigen for HI or MN assay according to predominant endemic strain circulating in poultry in the region surveyed. For specific strain information, please see the method and materials part of the corresponding reference

^b^Sera samples were collected from variety of mink populations during specific period. For the detailed information, please see the method and materials part of the corresponding reference

## Measures for blocking the interspecies transmission in mink

High susceptibility and possession of receptors to both avian and human influenza viruses fulfill the prerequisites for mink to severe as an intermediate host for interspecies transmission of influenza virus. A high level of seropositivity against diverse influenza strains in mink suggests the prevalence of the influenza virus, although outbreak reports are still limited. With the rapid increasing of fur-oriented mink industry, mink population is expected to expand. This increase in population may lead to the generation of novel reassortant with pandemic potential as a result of asymptomatic and mild infections with avian, human or swine influenza.

The systematic influenza surveillance network worldwide has significantly benefited the pandemic preparedness. However, more surveillance studies on mink farms are urgently needed due to their emerging role in the ecology of influenza virus. Regular virologic and sero-surveillance of influenza A virus should be implemented to monitor the health status of the mink. Specifically, diseased samples diagnosed with type A influenza virus should be carefully examined to subtype the HA and NA. The genetic information of the mink virus should be directly sequenced without egg culture and shared in a timely manner with the scientific community. As antigenic distant variants are co-circulated in poultry within large geographic regions in China, it will be important to select appropriate antigens for serologic survey. In addition, precautionary measures should be taken to enhance bio-security practises. Mink feed containing raw poultry or swine by-products should be accessed and examined to disinfect those with influenza infection. Farm workers should wear masks all times and minimize direct contact with minks during farming practises.

The most effective way to prevent zoonosis from triggering a pandemic was to control them at the source. Vaccination has been proved to successfully prevent and control infections. While mink vaccines against MEV, CDV, and hemorrhagic pneumonia have been widely used in China, the lack of vaccines against influenza may leave a pathway for influenza virus to become a pandemic. Avian influenza variants have a high potential to adapt well in mink and pose a further risk to human health. Immunization against influenza virus in mink population should be proposed in a timely manner. Bi-directional transmissions of SARS-CoV-2 between human and mink have necessitated the vaccination of mink to curb new waves of pandemic in human [47]. Thus, in line with the One Health concept, immunization of mink with the influenza vaccine could benefit not only the fur-oriented mink industry, but also public health.
